# Beyond satiety: unraveling the complex roles of POMC neurons in behavior and metabolism

**DOI:** 10.1007/s11154-025-09993-2

**Published:** 2025-09-19

**Authors:** Victor Jouque, Cristina Miralpeix, Antonio J. López-Gambero, Jean Charles Nicolas, Carmelo Quarta, Daniela Cota

**Affiliations:** University of Bordeaux, INSERM, Neurocentre Magendie, U1215, Bordeaux, F-33000 France

**Keywords:** POMC neurons, Energy balance, Hypothalamus, Food intake, Behavior, Obesity

## Abstract

Hypothalamic pro-opiomelanocortin (POMC) neurons are classically viewed as mediators of satiety, acting in response to metabolic and hormonal cues and in opposition to Agouti-related protein (AgRP) neurons to maintain energy balance. This model, centered on the appetite-suppressant effects of the POMC-derived neuropeptide α-melanocyte-stimulating hormone (α-MSH) through its activation of melanocortin-4 receptors (MC4R), has shaped our understanding of feeding and body weight regulation for decades. However, recent discoveries have challenged and expanded this traditional view, revealing that POMC neurons are not a uniform population dedicated solely to satiety control. Single-cell transcriptomic analyses have revealed striking molecular heterogeneity, reflected in distinct anatomical distributions, receptor expression profiles, electrophysiological properties, and projection patterns — all supporting the idea of functional specialization within this neuronal population. In this review, we propose a conceptual framework that integrates POMC neuronal heterogeneity with the regulation of appetite, metabolic physiology, and behavior beyond feeding. We highlight emerging evidence showing that discrete POMC neuronal subpopulations respond to specific combinations of interoceptive and environmental cues to orchestrate diverse adaptive responses. This perspective underscores the developmental plasticity and functional versatility of POMC neurons, offering new insights into the mechanisms of obesity and potentially paving the way for novel targeted therapeutic strategies.

## Introduction

In 1994, the discovery of the adipokine leptin revolutionized our understanding of energy homeostasis and triggered a wave of research into the neuroendocrine regulation of feeding behavior and metabolism [1–3].This breakthrough led to the identification of key hypothalamic circuits, particularly within the arcuate nucleus (ARC), that integrate metabolic signals to regulate energy balance. Among these circuits, the melanocortin system emerged as a critical pathway governing appetite, energy expenditure, and whole-body metabolism [4]. At the heart of this system there are two neuronal populations located in the ARC that exert opposite effects on energy balance through the release of specific neuropeptides acting on melanocortin receptors [5]. After a meal, pro-opiomelanocortin (POMC) neurons release α-melanocyte-stimulating hormone (α-MSH) from the cleavage of the POMC peptide. In turn, α-MSH activates the melanocortin 4 receptors (MC4R) in target brain regions, promoting satiety and increasing energy expenditure [6, 7]. Conversely, energy deprivation activates a second neuronal population co-expressing agouti-related peptide (AgRP) and neuropeptide Y (NPY). AgRP acts as an inverse agonist of MC4R, blocking α-MSH signaling to stimulate food intake and reduce energy expenditure [8, 9]. AgRP/NPY neurons also directly inhibit POMC neurons by releasing NPY and GABA [10].

This simplified model, portraying POMC and AgRP neurons as yin and yang partners in the control of food intake, has long served as a framework to understand the regulation of energy balance, also supported by the discovery that loss-of-function mutations in POMC and MC4R are the most common causes of monogenic obesity in humans [11, 12]. Hence, decades of research have established POMC neurons as satiety-promoting cells, receiving diverse inputs from multiple brain regions, and acting as central hubs where hormones, circulating nutrients, and gut-derived peptides are translated into neural responses via extensive projections throughout the hypothalamus and forebrain regions [13, 14].

However, recent evidence suggest that POMC neurons exhibit greater functional diversity than previously recognized, challenging the traditional view of POMC neurons as exclusive drivers of satiety [15–19]. This functional complexity may reflect an ancient evolutionary heritage, since the *POMC* gene emerged over 500 million years ago, during a time when food availability was unpredictable, and survival depended on the ability to balance the obtention of food with the risk of exposure to threats. Hence, the diversification of *POMC* gave rise to a multifunctional protein precursor capable of modulating a broad range of physiological responses essential for survival [5, 20, 21]. The missing piece of the puzzle in fully understanding what POMC neurons do may lie in the discovery that they display striking molecular heterogeneity. Thus, molecular diversity likely underlies functional specialization, with distinct POMC neurons subpopulations regulating specific components of energy balance and beyond, including glucose homeostasis, thermoregulation, pain, stress responses, and reward processing [5, 22–24].

In this review, we highlight the latest advances in unraveling the complex roles of hypothalamic POMC neurons in physiology and pathology, providing new insights into how they contribute to feeding behavior, metabolic regulation, and broader behavioral processes, hence offering a deeper understanding of the pathophysiology of obesity and potential new therapeutic avenues.

### Discovery of POMC neurons as key mediators of satiety: from past to present

The cloning of the POMC gene in 1978 was a major milestone in neuroendocrinology and beyond, revealing a single precursor polypeptide capable of generating a range of bioactive peptides, including α-MSH, adrenocorticotropic hormone (ACTH), and β-endorphin [25, 26]. POMC is functionally expressed in the ARC and nucleus of the solitary tract (NTS) of the brainstem, pituitary and skin [13]. The first POMC cDNA was cloned from bovine pituitary, followed shortly after by its human and murine counterparts, underscoring the evolutionary conservation of this gene across mammals and its physiological importance [13]. Initially, α-MSH was studied for its role in pigmentation through its effects on MC1R in skin melanocytes. A first study in 1976 by Panskepp and colleagues suggested that the peptide could decrease food intake [27], which was confirmed by additional investigations in the 1990 s [13]. MC4R, a G-protein-coupled receptor highly expressed in the hypothalamus and brainstem among various other brain regions, was then cloned in 1992 and found to mediate the anorexigenic effects of α-MSH [28]. Then mutations in MC4R emerged as the most common monogenic cause of early-onset obesity in humans, underscoring its critical role in appetite regulation [9]. In parallel, loss-of-function mutations in POMC and other genes related to the melanocortin pathway caused hyperphagia and obesity in both rodents and humans, reinforcing the idea that POMC neurons are key mediators of satiety [11, 12]. Accordingly, pharmacological stimulation of hypothalamic MC4R by intracerebroventricular (ICV) administration of an α-MSH analog led to a robust reduction in food intake [29, 30]. Conversely, pharmacological MC4R antagonism blocked this effect, whereas the administration of AgRP or the MC4R antagonist SHU9119 alone promoted hyperphagia [31, 32]. Similarly chronic silencing of POMC neurons [33] or POMC neuronal ablation causes hyperphagia and massive obesity [34, 35], while their optogenetic activation suppresses food intake through MC4R signaling [36]. These findings also spurred intense efforts to develop MC4R agonists as potential anti-obesity treatments, leading to the synthesis of new α-MSH analogs, such as setmelanotide, which is being used to treat genetic obesity syndromes [27].

While most early studies focused on the hypothalamic POMC neurons in the ARC, a second, smaller population of POMC neurons exists in the NTS where it integrates gut-derived satiety hormones, such as cholecystokinin (CCK) and glucagon-like peptide-1 (GLP-1). Through their intra-brainstem projections, NTS-POMC neurons acutely reduce food intake, likely through MC4R signaling controlling vagal output and gastric motility [37–39] and complement the actions of the ARC-POMC neurons, creating a multi-level regulatory system that ensures that food intake is finely adjusted to the body’s needs.

### POMC neuronal heterogeneity: one population, multiple POMC neurons subtypes

POMC neurons have been historically recognized as a single neuronal population because of the expression of the POMC peptide. However, a major and relatively recent advance in the understanding of POMC neuronal biology has been the recognition of the extensive molecular heterogeneity of POMC neurons [40], which was further confirmed by high-throughput single-cell RNA sequencing (scRNA-seq) techniques developed in recent years [22–24]. A detailed transcriptomic analysis of lineage-traced POMC-expressing cells during brain development revealed that these neurons form specific subpopulations [41]. This was further confirmed by studies revealing that this heterogeneity persists in adulthood, encompassing differences in gene expression, anatomical distribution, receptor profiles, electrophysiological properties, neurotransmitter identity, and projection sites [5, 24].

#### Molecular heterogeneity

ScRNA-seq have revealed that only 10% of ARC POMC neurons express Glucagon-like peptide 1 receptor (GLP1R), 25% express Leptin receptor (LepR), 64% express Insulin receptor (InsR) and they do not necessarily overlap. 36% of POMC neurons lack both LepR and InsR, indicating that certain subsets may be insensitive to these hormonal cues. This strong molecular diversity may underlie the observation that only 20–40% of POMC neurons exhibit activity changes in response to nutritional challenges, as assessed by c-Fos expression [42–45]. Accordingly, the molecular Heterogeneity in the expression of receptors is reflected in the different impact of their deletion on physiology. For example, postnatal ablation of serotonin receptor 5-HT2C in POMC neurons promotes hyperphagia and obesity under obesogenic diet [46], while postnatal ablation of LepR in POMC neurons disrupts systemic glucose control and impairs leptin production from the adipose tissue, without altering body weight or feeding behavior [47].

Additionally, 27% of POMC neurons co-express AgRP and NPY in adulthood, raising the hypothesis that these neurons could behave similarly to AgRP neurons and exert orexigenic effects under specific conditions. Interestingly, this cluster of POMC neurons is characterized by a low expression of the key identity POMC markers. By then using genetic lineage tracing in adult animals, we identified subpopulations described as “ghost POMC neurons”, exhibiting negligible POMC expression and displaying atypical molecular and functional identity [46]. These atypical POMC cells show reduced responsiveness to nutritional challenges and to leptin and insulin, and have decreased expression of LepR, InsR and GLP1R [46]. Intriguingly, Ghost neurons were recruited in a mouse model of adult-onset obesity, independent of neurogenesis or changes in neuronal turnover [46]. One interpretation of these findings is that subsets of typical POMC neurons may undergo shifts in their functional identity, leading to changes in the expression of the molecular machinery that enables responses to peripheral metabolic signals following exposure to obesogenic stimuli in adulthood, and hence accumulation of atypical Ghost neurons [46]. The exact role of Ghost neurons in both physiology and pathology is still not well understood. To clarify their functional significance, it will be important to identify molecular markers that are uniquely expressed by this specific group of neurons. Additionally, developing transgenic mouse models that allow for the selective targeting of Ghost neurons versus canonical POMC neurons will be crucial. Using then intersectional genetic strategies [47–49], which have been effective in studying different subpopulations of POMC neurons in vivo could aid in this investigation [48, 50]. Hence, POMC neurons may exhibit heterogeneous functional responses, linked to the differential expression of markers of functional identity, including hormones receptors, neurotransmitters receptors and POMC itself [48, 51].

POMC neurons can also be segregated into four subpopulations based on their expression of GABA, glutamate, both or none of these 2 neurotransmitters [52]. These clusters exhibit distinct molecular profiles, raising the question of whether they may be involved in different functions related to energy balance, metabolic regulation, and beyond [53]. While most data come from rodent models, recent single-nucleus RNA-seq studies in human postmortem hypothalamus indicate that the principle of POMC neuronal heterogeneity is largely conserved across species [54]. Cross-species transcriptomic alignment showed that human POMC clusters map onto mouse counterparts, indicating conserved molecular identities, while also revealing human-specific adaptations in gene expression and receptor signaling. Notably, in humans, POMC clusters co-express GLP1R and LepR within the same populations, whereas these receptors mark separate subsets in mice, highlighting species-specific organizational differences. Besides, the third human POMC subcluster (C4-375) matched the GLP1R POMC cluster, albeit with low correlation, indicating divergence in this neuronal subtype. Additionally, the human C4-374 cluster, corresponding to mouse Glp1r-expressing POMC neurons, instead expresses the calcitonin receptor (CALCR), highlighting species-specific adaptations in receptor expression with potential implications for obesity therapies.

Together, these results demonstrate that the principle of POMC neurons molecular heterogeneity is conserved across species, while also underscoring critical species-specific differences that need to be considered when extrapolating rodent findings to humans.

#### Spatial heterogeneity and projection sites

The spatial positioning of POMC neurons within the ARC, located close to the median eminence, which lacks a blood-brain barrier and provides easier access to circulating signals, may influence the temporal dynamics of hormonal responses. Accordingly, the peripheral administration of leptin activates leptin-sensitive neurons along the ventro-dorsal axis of the hypothalamus at different time scales [55]. Besides, LepR-, GLP1R- and InsR-responsive POMC neurons display segregated distributions across the ARC, with LepR-expressing POMC neurons found throughout the ARC, GLP1R-expressing POMC neurons predominantly located in the caudal ARC and InsR-positive POMC neurons largely found in the rostral part of the ARC [48, 56]. Beyond receptor-based spatial segregation, neurotransmitter profile also correlates with anatomical distribution, with GABAergic POMC neurons positioned closer to the third ventricle, while glutamatergic POMC neurons are situated more laterally [53]. This spatial heterogeneity across both hormone receptor expression and neurotransmitter content indicates that POMC neurons are not only molecularly diverse but also anatomically organized, potentially enabling them to mediate different functions depending on their location within the ARC. The exact reason for the rather segregated spatial distribution of POMC neurons subpopulations remains unknown. One possibility is that the proximity to the median eminence and ease of exposure to diffused nutritional cues might have evolutionarily determined POMC neurons subpopulations distribution [5]. In support of this, perineuronal nets (PNNs)—specialized extracellular matrix structures—are enriched in the ARC, likely facilitating selective access of blood-borne signals to ARC neurons. Recent findings have shown that PNNs modulate the permeability of this area and regulate the exposure of ARC neurons to circulating metabolic cues [57–61]. Notably, only ~ 30–50% of ARC POMC neurons are encapsulated by PNNs, raising the possibility that PNN-dependent filtering of peripheral signals contributes to the functional heterogeneity observed within this population [61]. Although direct experimental evidence for the role of PNNs in modulating the function of POMC neurons remains limited, it has been shown that modifying PNNs can alter AgRP neurons fibre density and cell numbers [62], as well as their responsiveness to metabolic signals [60], implying that similar manipulations could affect POMC neurons.

Spatial distribution of POMC neurons within the ARC is then accompanied by differences in projections to downstream targets [63]. Tracing studies have demonstrated that only about 8% of POMC neurons project to the paraventricular nucleus of the hypothalamus (PVN), with the majority of these PVN-projecting neurons located in the mid-to-caudal portion of the ARC [64]. POMC cells located in the rostral part of the ARC predominantly project to autonomic regions, whereas caudal POMC neurons primarily project to hypothalamic nuclei [14]. This distinction in projections patterns suggests that POMC neuronal clusters projecting to different brain regions may originate from specific subdomains within the ARC, likely reflecting differences in their physiological roles.

#### Electrophysiological heterogeneity

Several studies have also demonstrated that molecularly distinct POMC neurons exhibit diverse electrophysiological properties. For example, 30% of POMC neurons increase firing in response to leptin, and this response does not overlap with POMC cells in which insulin modulates firing [56]. Similarly, LepR- and Glp1R-expressing POMC neurons not only differ in their molecular signatures but also show markedly distinct electrophysiological phenotypes, reflecting their differential roles in the regulation of food intake [48]. POMC glutamatergic and GABAergic neurons can also be electrically distinguished. Interestingly, these two subpopulations display opposite responses to the inhibition of the mechanistic target of rapamycin Complex1 (mTORC1), a key cellular energy sensor in the hypothalamus, further highlighting their functional divergence [53, 65]. Additionally, under conditions of glucose deprivation, some POMC neurons demonstrate increased electrical activity, while others show a decrease in firing rate or a biphasic response [66]. However, molecular diversity does not always translate into electrophysiological differences. For example, both 5-HT2C- and LepR-expressing POMC neurons share a common intracellular depolarization mechanism, involving the activation of the putative transient receptor potential C5 (TRPC5) channels [67, 68]. Despite this common mechanism, these two subpopulations have different Functional outputs, as 5HT2C-expressing POMC neurons are directly involved in feeding behavior, whereas LepR-expressing POMC neurons are not [69, 70].

Collectively, these findings highlight the remarkable heterogeneity of POMC neurons, challenging the long-held view of POMC neurons as a uniform population dedicated solely to promoting satiety, and supporting the idea that POMC neurons may have complex roles in energy balance regulation and beyond.

### How is heterogeneity established? ontogeny of POMC neurons

The ARC of the hypothalamus contains only around 9000 POMC-expressing neurons in rodents [71]. Yet, despite their relatively small number, these neurons orchestrate a vast array of complex functions, regulating a wide range of metabolic and hormonal responses. Given that POMC neurons emerge earlier than AgRP and give rise to AgRP and other hypothalamic neuronal populations [72], understanding their neurodevelopmental plasticity is crucial for uncovering early determinants of metabolic control and metabolic disease.

*Pomc* expression begins at embryonic day 10.5 in the developing ventral hypothalamus. During gestation, certain *Pomc*-expressing cells can switch off POMC production, giving rise to NPY/AgRP neurons or other cell types, including Kisspeptin neurons, which are sets of neurons in the ARC expressing and releasing the neuropeptide Kisspeptin to activate gonadotrophin-releasing hormone (GnRH) neurons to which they project [73, 74]. The ontogeny of POMC and other ARC neurons is driven by transcription factors, such as Distal-less homeobox-1/2 (Dlx1/2), Orthopedia (Otp), and neurogenin 3 [75, 76], with additional regulation by microRNAs and other epigenetic mechanisms [77, 78]. A combination of spatial and temporal cues is crucial for regulating POMC neuronal differentiation, migration, and maturation. In this context, additional key transcription factors, including Nkx2.1, Rax, and Tbx3 [44, 79, 80], activate the expression of POMC in neurons and therefore play essential roles in specifying POMC neuronal fate. Besides, axon guidance cues, including the axon guidance protein Netrin-1 and Slit/Robo protein complex, also play key roles in establishing proper projections between POMC neurons and downstream targets [72, 81]. Advancements in scRNA-seq have provided further information on developmental trajectories of hypothalamic POMC neurons, identifying distinct subpopulations characterized by unique neuropeptide expression profiles and molecular signatures early on, which continue to mature postnatally giving raise to distinct adult subpopulations [41]. This evidence suggests that the heterogeneity and plasticity of POMC neurons is strongly controlled by developmental mechanisms, which however may provide this population of neurons with a degree of plasticity that is maintained into adulthood. Accordingly, it has been proposed that subsets of POMC neurons may undergo shifts in functional identity in response to an obesogenic diet in adulthood, leading to changes in the molecular machinery that mediates responses to metabolic signals [51]. Beyond these Lineage-specific transcriptional programs, and as previously mentioned, also epigenetic mechanisms play a crucial role in shaping POMC neurons identity and function through development. For instance, using a human embryonic stem cell model, Lechner and colleagues showed that reduced POMC gene expression was associated with increased POMC methylation in POMC-expressing neurons and they identified an epigenetic obesity risk variant at the POMC gene, which carries a 1.4-fold increased risk of developing obesity in humans [82]. Some of the molecular mechanisms regulating POMC neurons development are also sexually dimorphic. This is the case for the transcriptional regulator Six3, whose absence reduces *Pomc* expression, and leads to hyperphagia and mild obesity in male but not in female mice [83].

In addition, endocrine signals, such as leptin, insulin, and glucocorticoids, as well as environmental factors, including maternal nutrition, play crucial roles in regulating POMC neuron differentiation, survival, and function [5]. Maternal exposure to high-fat diet during lactation impacts early postnatal neurogenesis and expands the NPY to POMC neuronal ratio, establishing an obesogenic tone [84, 85]. Accordingly, altered *Pomc*, *Agrp*, and *Kiss* mRNA levels are observed in pups with maternal metabolic stress [86–88]. Moreover, POMC neuronal projection profiles are altered in response to maternal obesity [89, 90], and are accompanied by reprogramming of molecular pathways controlling axonal growth [91]. Altered levels of hormones such as insulin and leptin are then thought to contribute to cellular reprogramming of POMC neurons following maternal obesity. For instance, insulin signaling in POMC neurons is crucial for proper neural projections, as its alteration due to maternal overnutrition leads to defects in POMC connectivity to the PVN, pancreatic parasympathetic innervation, and glucose-stimulated insulin secretion [92].

Collectively, these findings support the notion that altered programming of the neural network, beginning as early as embryonic development and driven by changes in upstream and downstream signals, regulates POMC neuronal development via sex-dependent mechanisms, ultimately affecting POMC neurons function and susceptibility to metabolic disease later in life. In addition to developmental mechanisms, numerous studies using genetic mouse models have investigated the roles of several intracellular mechanisms, spanning from potassium channels to autophagy, ER-stress and specific signaling pathways engaged by hormones, cytokines and nutrients, often revealing important roles for the correct function of POMC neurons and the consequent impact on the development of obesity. However, some of these studies are limited by the genetic approaches used, as constitutive deletion of the molecular target investigated may impact on neuronal development and/or touch upon neuronal groups that are not POMC neurons but briefly express *Pomc* during development. The reader is referred to previous recent reviews where we have provided greater details upon these studies [5, 93].

### Dismantling the old vision of POMC neurons as exclusive mediators of satiety

A key observation challenging the view of ARC POMC neurons as exclusive mediators of satiety came from chemogenetic studies showing that the activation of this neuronal population must be sustained for several hours to effectively suppress food intake. In contrast, activation of NTS POMC neurons leads to an immediate inhibition of feeding [94]. This suggests that POMC neurons may operate on distinct time scales, integrating different signals and/or engaging different circuits, going beyond the typical paradigm of POMC versus AgRP neurons. Importantly, in vivo calcium imaging studies have shown that ARC POMC neurons become active as soon as food is available and in response to food-associated, sensory cues, even before food consumption [15]. Thus, POMC neurons not only encode satiety signals following food intake, but also respond to anticipatory processes of food ingestion, priming peripheral organs like the liver to the arrival of the meal [16]. This process involves modifications in hepatic mitochondrial dynamics facilitating insulin-induced suppression of hepatic glucose production, which is crucial for metabolic adaptation after a meal [16].

Furthermore, recent chemogenetic and optogenetic studies have demonstrated that hypothalamic POMC neurons can stimulate feeding under specific conditions. For example, cannabinoid-induced hyperphagia depends on the activation of cannabinoid type-1 receptors (CB1R) on POMC neurons, which triggers the release of the POMC-derived peptide β-endorphin, rather than α-MSH [18]. In line with this, optogenetic activation of POMC neurons projecting to the paraventricular thalamus (PVT) specifically promotes sugar overconsumption through an opioidergic circuit, involving µ-opioid receptor (MOR) activation by β-endorphin [19]. Thus, potentially distinct POMC neuronal subpopulations can release different neuropeptides with opposing functions, depending on metabolic and environmental cues, likely modulating specific projection sites, although the molecular mechanisms underlying the release of different POMC-derived neuropeptides remain to be elucidated.

Another key factor contributing to the functional heterogeneity of POMC neurons is their neurotransmitter profile, as mentioned earlier, and specifically their classification in GABAergic and/or glutamatergic cells [52]. Mice with a mutation in the *Pomc* gene develop obesity. Remarkably, selective postnatal re-expression of *Pomc* in POMC-GABAergic neurons reduces food intake and fully reverses the obese phenotype [95]. Surprisingly, non-selective re-expression of *Pomc* in all POMC neurons has negligible anti-obesity effects. Thus, in the absence of POMC, POMC-GABAergic neurons may promote feeding and weight gain through mechanisms that are yet to be understood. Consistently, chronic electrical activation of non-AgRP GABAergic neurons in the ARC leads to obesity [96].

In line with the previous findings, acute pharmacological inhibition of mTORC1 by rapamycin, which mimics a fasting-like state [65], activates POMC-GABAergic neurons while inhibiting POMC-glutamatergic neurons, implying that certain POMC neurons are activated in response to stimuli informing about a negative energy state, thereby promoting food intake [53]. Conversely, other POMC neuronal clusters, such as the glutamatergic ones, may mediate opposing effects, including satiety and energy expenditure, which are classically attributed to POMC neurons. Interestingly, “classical” POMC clusters have already been identified, such as the dopamine receptor 2 (Drd2)-expressing POMC neurons, which exhibit a specific translational profile and acutely suppress food intake while stimulating energy expenditure [50].

**Fig. 1:**
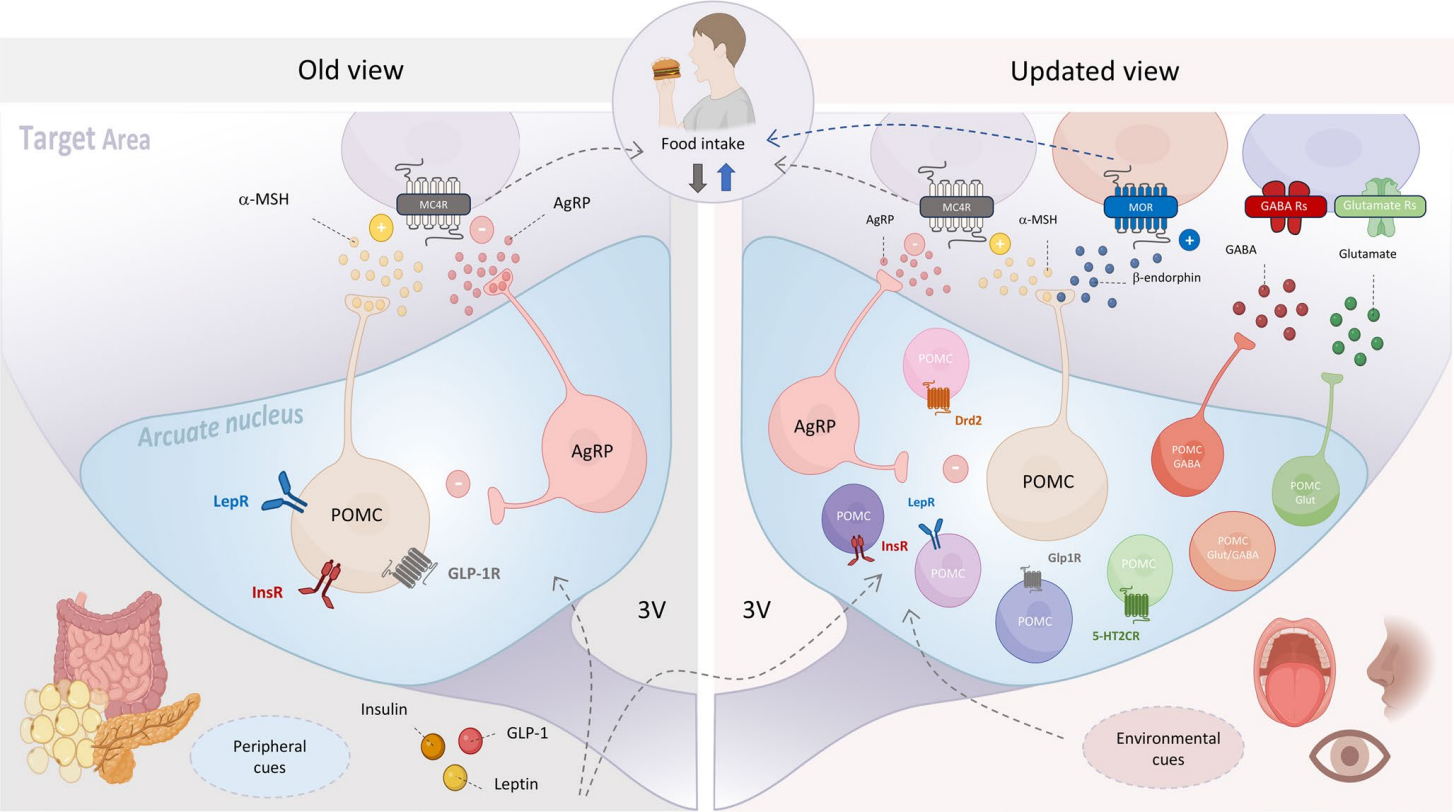
Old and updated view of hypothalamic POMC neurons in the regulation of food intake. Left panel: Traditional model depicting the yin-yang actions of the melanocortin system on feeding behavior. POMC neurons respond to postprandial hormonal signals by releasing α-MSH, which activates MC4R signaling to induce satiety. Conversely, AgRP neurons are activated during energy deprivation and release AgRP, an endogenous MC4R antagonist, to promote food intake. Right panel: Recent advances have revealed that POMC neurons consist of multiple, non-overlapping subpopulations characterized by distinct hormonal sensitivities, neurotransmitter phenotypes, and expression of specific receptors. In addition to responding to internal metabolic cues, some POMC neurons are activated by sensory signals prior to consumption and can promote feeding through release of β-endorphin 3 V: third ventricle; Glut: glutamate; MOR: µ-opioid receptor; GABA Rs: GABA receptors; Glutamate Rs: glutamate receptors; LepR : Leptin receptor; InsR : Insulin receptor; GLP-1R : GLP-1 receptor; Drd2 : Dopamine receptor 2; 5-HT2CR : 5-HT2C serotonin receptor

The increased use of intersectional strategies has further facilitated the functional characterization of several POMC neurons subpopulations, particularly based on their hormonal responsiveness. Notably, it has been shown that LepR-expressing and GLP1R-expressing POMC neurons differentially regulate food intake. Chemogenetic activation of GLP1R-expressing POMC neurons induces a stronger suppression of feeding compared to LepR-expressing POMC neurons, despite the fact that these two non-overlapping subpopulations share similar projection patterns, implying that modulation of underlying circuits is nuanced based on the hormonal input received [48]. In Fig. 1 we provide a simplified overview illustrating the evolution of our understanding of the role of ARC POMC neurons in the modulation of food intake.

### POMC neurons in the regulation of energy expenditure and metabolic responses

Appetite regulation does not fully cover the whole spectrum of the role of POMC neurons in energy balance. Early studies showed that ICV injection of the synthetic α-MSH analog melanotan II in rats triggered fat lipolysis and thermogenesis, accounting for weight loss compared to pair-fed rats [97]. Accordingly, investigations using mice models with global loss of hypothalamic POMC neurons described reduced energy expenditure accompanying the hyperphagic phenotype [34, 44, 94]. Further research in rodents has established a role for POMC actions via MC4R expressing neurons in driving brown adipose tissue (BAT) thermogenesis, white adipose tissue (WAT) browning, lipolysis and fat mobilization through activation of the sympathetic nervous system (SNS) [98–102]. This effect mainly involves β3-adrenergic receptors which highly innervate the BAT and WAT and respond to norepinephrine release upon SNS stimulation. Understanding the neurocircuitry involving ARC POMC neurons-mediated control of energy expenditure queries the target areas expressing MC4R that connect to the SNS. Retrograde viral tracing has identified several hypothalamic areas that couple with sympathetic terminals in the BAT and that express MC4R, such as the PVN, dorsomedial nucleus (DMN) and lateral hypothalamic area (LHA) [103]. MC4R-expressing PVN and DMN neurons are innervated by POMC neurons projections and their activation results in strong sympathetic activity and BAT thermogenic response [104–106]. It has been demonstrated in subsequent studies that MC4R in cholinergic preganglionic sympathetic neurons in the brainstem, mainly in the NTS, dorsal nucleus of the vagus nerve (DMV) and intermediolateral medulla also contribute to MC4R-mediated thermogenesis in the BAT [107]. POMC neurons also have collateral projections to BAT and iWAT [99], implying that adipose tissue thermogenesis may be indirectly and directly regulated by POMC neurons, although this overlapping connectivity is currently of unknown physiological significance. Specific subsets of POMC neurons may also regulate WAT Lipolysis via intestinal sympathetic nerves promoting bile acid release and activation of the bile acid receptor Takeda G-protein receptor 5 (TGR5) in the WAT, bypassing direct adipose sympathetic afferent input [108]. Research has also pointed out to alternative mechanisms for POMC neuron-mediated BAT thermogenesis. For instance, tyrosine-releasing hormone (TRH) is abundantly expressed in MC4R neurons in the PVN, but no evidence of alterations of thyroid hormones has been observed in MC4R-deficient mice and humans [85, 93, 99]. Nonetheless, deletion of the leptin-negative regulator Protein tyrosine phosphatase 1B (PT1B) in POMC neurons enhances POMC neuron-mediated cold-induced thermogenesis via elevated T3 levels. Hence, POMC neurons activity might be required for activation of the thyroid axis in response to a hypothermic stimulus [109], which is accompanied by cold-induced POMC-mediated increases in sympathetic activity [101].

Data on the role of POMC neurons in energy expenditure extend mostly to murine studies assessing action through MC4R. However, and opposed to constitutive deletion of *Mc4r* in mice, patients and animal models with *MC4R* mutations show no basal differences in energy rate [110–112], although administration of Setmelanotide, a full MC4R agonist, to patients with obesity increases resting energy expenditure by 6.4% as compared to placebo [113]. This suggests that constitutive MC4R impairment in humans has less impact on the peripheral control of energy expenditure relative to mice, while its activation recapitulates enhanced thermogenic activity. Although much less common than *MC4R*, *MC3R* polymorphisms and mutations in humans are associated with greater adiposity yet decreased lean mass and altered peripheral energy partitioning [114–116]. These metabolic traits are reproduced in mouse models of *Mc3r* deficiency, that depict either normal or hypophagic phenotypes with increased locomotion and energy expenditure [117–119]. Combined *Mc3r* and *Mc4r* deficiency further exacerbates the obese phenotype observed by *Mc4r* deficiency alone, as *Mc3r* deficiency enhances food efficiency and storage of fat [117]. In addition, *Mc3r* deficiency results in an enhanced anabolic response to a high-fat diet, and a higher anorexigenic response to caloric restriction or pharmacological activity of the MC4R agonist [119, 120]. Furthermore, the specific deletion of *Mc3r*-expressing neurons in the DMN, which contains large POMC neurons projections, decreases energy expenditure [119, 121]. Altogether, this evidence suggests that POMC neurons-mediated regulation of energy expenditure results from a complex interaction of cellular targets through the additive actions of both MC4R and MC3R.

Beyond the regulation of food intake, body weight and energy expenditure, POMC neurons directly respond to certain types of fatty acids and control hepatic glucose production, insulin sensitivity, glucose and lipid metabolism, illustrating their critical role in overseeing whole body use of energy substrates and consequent involvement in metabolic disorders [98, 122–126]. As mentioned earlier, distinct subpopulations of POMC cells are characterized by the expression of the *Lepr*, *Insr*, *Glp1r* and *5HT2Cr*, depicting different responsiveness to the related hormonal or neurotransmitter ligands [22, 24, 48, 56, 67, 68]. Deletion of *Lepr* in POMC neurons alone alters energy expenditure, glucose tolerance and hepatic insulin sensitivity, without affecting body weight [123, 127], whereas *Insr* deletion in POMC cells causes insulin insensitivity in the adipose tissues suppressing lipolysis, but without affecting energy balance [128]. Combined deletion of both *Lepr* and *Insr* in POMC cells further affects glucose homeostasis and insulin sensitivity [129], and the opposite is observed in transgenic mice with enhanced *Lepr* and *Insr* signaling in POMC neurons, showing heightened energy expenditure and glucose handling [130]. Conversely, mice with constitutive and postnatal ablation of *Htr2c* in POMC neurons are hyperphagic but retain functional energy expenditure responses [69], whereas *Glp1r* deletion in POMC neurons has only subtle effects on weight gain in high-fat diet [131]. Altogether, these studies clearly highlight that the diverse molecular subsets of POMC neurons responsive to different hormonal cues guide adaptative physiological responses to different metabolic needs.

Finally, it should also be mentioned that aging is characterized by changes in energy expenditure and lipid and glucose metabolism. Aging is associated with a decline in both cellular function and the whole-body’s ability to use and/or store energy reserves, which has been referred as “metabolic aging” [132, 133]. Intriguingly, in aging mice, hypothalamic neurons are particularly susceptible to molecular hallmarks of aging [134], highlighting this brain region as a critical hub of metabolic aging. Increased evidence points towards a reduced activity of POMC neurons as a contributing factor for these age-related impairments. Aged mice have reduced POMC activity [135], whilst overexpression of POMC in aged rats improves fat metabolism and glucose tolerance [136]. Elevated mTORC1 signaling, which is strongly associated with age-dependent obesity, is observed in POMC neurons and drives diminished POMC neuronal firing with decreased sympathetic tone and lipid metabolism, which is reversed by the mTORC1 antagonist rapamycin [108, 135, 137]. Age-related loss of autophagy capacity in POMC neurons is also linked to reduced α-MSH levels and ultimately causes impaired glucose tolerance and peripheral lipolysis in response to food deprivation [138]. Additionally, AgRP innervation onto POMC neurons is increased with age and recapitulates neuronal adaptation to a high-fat diet [139]. Hence, a deeper understanding of the impact of age-related changes on the functions of POMC neurons is necessary to address future challenges for the treatment of obesity in an increasingly aging population.

### POMC neurons beyond energy balance and metabolism

**Fig. 2:**
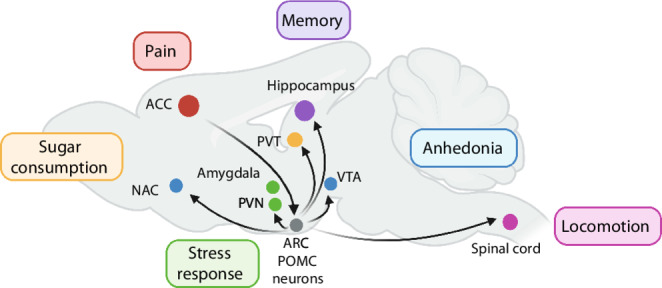
Main brain targets of hypothalamic POMC neurons in the regulation of behaviors beyond energy balance. POMC neurons have been described to regulate various behavioral processes necessary for survival, spanning from locomotion, and stress response to pain. Regulation of emotional responses by these neurons also impacts on feeding PVN: Paraventricular nucleus of the hypothalamus; NAC: nucleus Accumbens; PVT: Paraventricular nucleus of the thalamus; VTA: Ventral tegmental area; ACC: Anterior cingular cortex

In natural habits, animals must constantly adapt their behavior to resolve the conflict between internal energy state and external environmental perturbations [140]. For instance, the presence of a predator will rapidly modify eating behavior to prioritize self-preservation responses by recruiting hypothalamic neuronal circuits [141, 142]. As noted above, the heterogeneity of POMC neurons and their vast inputs and outputs projections sites imply a wide range of functions according to nutrients and hormonal sensing [5, 14] (see Fig. 2). This includes growing evidence suggesting that POMC neurons may regulate behaviors and responses beyond actual food consumption, which however are necessary for guarantying survival, while looking for and consuming food. As a result, and as briefly illustrated below, hypothalamic POMC neurons can modulate locomotion, responses to pain, and to stressful conditions, while also affecting memory processes.

Locomotion requires energy, yet animals need to increase locomotion to find and consume food. Hypothalamic POMC neurons have long-range projections into spinal cord regions harboring MC4R-expressing V2a interneurons, crucial components of the premotor networks. Acute inhibition of MC4R signaling in these interneurons reduces locomotor activity, implying a direct role for POMC neurons in the control of locomotion [143]. Besides, exercise activates hypothalamic POMC neurons [144, 145]. Changes in POMC neuron activity during exercise appear to be sensitive to the organism’s energy state, since exercise increases excitatory inputs onto LepR-expressing POMC neurons [127, 144, 145]. In addition, the beneficial metabolic effects of regular running exercise are mediated by mitochondrial adaptations that directly affect the function of hypothalamic POMC neurons by stimulating β-endorphin production [146].

It is therefore not surprising that, in addition to exercise, hypothalamic POMC neurons may also modulate the release of β-endorphin, a known analgesic, in response to pain. In turn, chronic pain has been shown to suppress feeding behavior through a specific brain circuit involving ARC POMC neurons [147]. Persistent pain activates glutamatergic neurons in the anterior cingulate cortex (ACC), which projects to the lateral hypothalamus and subsequently to POMC neurons in the ARC to ultimately activate these cells and suppress feeding [147]. Therefore, POMC neurons regulate both appetite suppression and chronic pain through specific neuronal circuits. What molecular pathways and subpopulation of POMC neurons are regulating pain-induced suppression of feeding remain, however, to be elucidated.

Like pain, stress can modify feeding behavior in rodent and humans and it can affect emotional responses and body weight [148]. Hypothalamic POMC neurons regulate both acute and chronic stress responses through distinct neuronal circuits [149–151]. Acute physical restraint stress activates ARC POMC neurons which in turn stimulate the hypothalamus-pituitary-adrenal axis by activating corticotrophin-releasing hormone (CRH) neurons in the PVN [63, 149, 152]. Interestingly, POMC neurons activation is stressor-specific as these neurons respond to acute restraint stress and forced swim test but not to predator odor exposure. In addition, activation of ARC POMC neurons shows variations along the rostro-caudal axis, highlighting that different subpopulations or circuits may mediate responses to distinct stressors [149, 152]. Chronic predictable and unpredictable stress also lead to hyperactivity of POMC neurons due to a reduction on the GABAergic inhibition and increased intrinsic excitability of POMC neurons [151, 153]. These neurons send inhibitory terminals to dopamine-producing neurons in the ventral tegmental area (VTA). Inhibition of the POMC^ARC^→DA^VTA^ circuit in chronically stressed mice reduces depression-like behaviors and anhedonia while increasing food intake [151, 153]. Instead, chemogenetic activation of POMC neurons is sufficient to induce anhedonia and behavioral despair under non-stress conditions [151]. Of note, the effects of chronic activation or inhibition of POMC neurons differ between sexes, since female mice show resilience to chemogenetic manipulations of ARC POMC neurons despite similar electrophysiological changes under stress [151]. In addition, alcohol consumption seems to preferentially activates specific subsets of hypothalamic POMC neurons projecting to the stress regulating areas such as the amygdala [154], and epigenetic modifications in the *Pomc* promoter region might partially explain the transgenerational effects of alcohol fetal exposure through early-life POMC molecular underpinning [155].

In humans, epigenetic modifications of the *Pomc* gene promoter (methylation at specific CpG sites) is associated with depressive disorders and self-injurious behaviors in adolescents [156] and certain genetic variations in *Pomc* gene appear to influence treatment outcomes for major depressive disorder in adults [157]. Clearly, however, in these studies, the expression and function of POMC is not only affected in the hypothalamus, but also for instance in the pituitary, importantly impacting on hormonal responses. Notwithstanding, it is worth mentioning that a recent study carried out on human hypothalamus showed that subjects died by suicide and suffering from mood disorder have greater expression of progesterone receptors on hypothalamic POMC neurons [158]. Interestingly, higher plasma progesterone concentrations have been reported in women with recurrent suicide attempts [159]. The broader literature nevertheless points to progesterone being more consistently associated with suicidal ideation rather than with attempts, although the available studies are still few and call for cautious interpretation [160]. How progesterone may impact on the function of hypothalamic POMC neurons remains at present unknown.

Collectively, these findings highlight a role of hypothalamic POMC neurons and their downstream circuitry in modulating responses to stress and emotional states, with a potential involvement in the susceptibility to mood disorders.

Finally, eating behavior is intimately connected with multiple and complex neuro-cognitive processes allowing the identification and association of different types of food with specific outcomes for survival. It is therefore unsurprising that recent evidence has shown a role for hypothalamic POMC neurons in the regulation of memory processes through the ability of these neurons to synthetize the neurosteroid precursor pregnenolone [161]. Effects of pregnegnolone on cognitive function were mediated via an autocrine mechanism on POMC neurons, influencing hippocampal long-term potentiation [161]. This mechanism was found to be altered in obesity and may therefore help explain the presence of cognitive disorders associated with obesity [161, 162].

## Conclusions

In summary, hypothalamic POMC neurons’ diversity in the anatomical and molecular dimensions and their ability to release different types of neuropeptides and neurotransmitters underlie their capacity to regulate multiple behaviors and metabolic responses, depending on the organism’s energy state and the specific environmental context. This heterogeneity helps explain the complex roles of POMC neurons in both driving and inhibiting the consumption of food as well as simultaneously regulating behaviors that might be in competition with feeding and that may influence feeding [163].

The evidence we have reviewed here clearly continues to point to hypothalamic POMC neurons as important targets for the treatment of obesity and metabolic disease. Current available literature also leads us to conclude that fully disentangling the actual biological functions of POMC neurons may help reveal the complex biological substrate linking metabolic disorders with psychiatric and neurodegenerative pathologies. However, given the heterogeneity of POMC neurons, selectively targeting individual subpopulations for therapeutic purpose remains challenging. Future approaches may require the identification of unique molecular markers, receptor combinations, or epigenetic signatures that allow highly selective targeting, potentially enabling the development of interventions that fine-tune energy balance while minimizing unintended effects on mood, stress, or other behavioral processes.

## Data Availability

No datasets were generated or analysed during the current study.

## References

[1] Friedman JM, Halaas JL. Leptin and the regulation of body weight in mammals. Nature. 1998;395:763–70. 9796811 10.1038/27376

[2] Montague CT, Farooqi IS, Whitehead JP, Soos MA, Rau H, Wareham NJ, et al. Congenital leptin deficiency is associated with severe early-onset obesity in humans. Nature. 1997;387:903–8. 9202122 10.1038/43185

[3] Clément K, Vaisse C, Lahlou N, Cabrol S, Pelloux V, Cassuto D, et al. A mutation in the human leptin receptor gene causes obesity and pituitary dysfunction. Nature. 1998;392:398–401. 9537324 10.1038/32911

[4] Balthasar N, Dalgaard LT, Lee CE, Yu J, Funahashi H, Williams T, et al. Divergence of melanocortin pathways in the control of food intake and energy expenditure. Cell. 2005;123:493–505. 16269339 10.1016/j.cell.2005.08.035

[5] Quarta C, Claret M, Zeltser LM, Williams KW, Yeo GSH, Tschöp MH, et al. POMC neuronal heterogeneity in energy balance and beyond: an integrated view. Nat Metab. 2021;3:299–308. 33633406 10.1038/s42255-021-00345-3 PMC8085907

[6] Cowley MA, Smart JL, Rubinstein M, Cerdán MG, Diano S, Horvath TL, et al. Leptin activates anorexigenic POMC neurons through a neural network in the arcuate nucleus. Nature. 2001;411:480–4. 11373681 10.1038/35078085

[7] Andermann ML, Lowell BB. Toward a wiring diagram understanding of appetite control. Neuron. 2017;95:757–78. 28817798 10.1016/j.neuron.2017.06.014 PMC5657399

[8] Ollmann MM, Wilson BD, Yang YK, Kerns JA, Chen Y, Gantz I, et al. Antagonism of central melanocortin receptors *in vitro* and *in vivo* by agouti-related protein. Science. 1997;278:135–8. 9311920 10.1126/science.278.5335.135

[9] Krashes MJ, Lowell BB, Garfield AS. Melanocortin-4 receptor–regulated energy homeostasis. Nat Neurosci. 2016;19:206–19. 26814590 10.1038/nn.4202 PMC5244821

[10] Tong Q, Ye C-P, Jones JE, Elmquist JK, Lowell BB. Synaptic release of GABA by AgRP neurons is required for normal regulation of energy balance. Nat Neurosci. 2008;11:998–1000. 19160495 10.1038/nn.2167 PMC2662585

[11] Krude H, Biebermann H, Luck W, Horn R, Brabant G, Grüters A. Severe early-onset obesity, adrenal insufficiency and red hair pigmentation caused by POMC mutations in humans. Nat Genet. 1998;19:155–7. 9620771 10.1038/509

[12] Huszar D, Lynch CA, Fairchild-Huntress V, Dunmore JH, Fang Q, Berkemeier LR, et al. Targeted disruption of the melanocortin-4 receptor results in obesity in mice. Cell. 1997;88:131–41. 9019399 10.1016/s0092-8674(00)81865-6

[13] Anderson EJP, Çakir I, Carrington SJ, Cone RD, Ghamari-Langroudi M, Gillyard T, et al. 60 YEARS OF POMC: regulation of feeding and energy homeostasis by α-MSH. J Mol Endocrinol. 2016;56:T157–74. 26939593 10.1530/JME-16-0014 PMC5027135

[14] Wang D, He X, Zhao Z, Feng Q, Lin R, Sun Y, et al. Whole-brain mapping of the direct inputs and axonal projections of POMC and AgRP neurons. Front Neuroanat. 2015;9:40. 25870542 10.3389/fnana.2015.00040 PMC4375998

[15] Chen Y, Lin Y-C, Kuo T-W, Knight ZA. Sensory detection of food rapidly modulates arcuate feeding circuits. Cell. 2015;160:829–41. 25703096 10.1016/j.cell.2015.01.033 PMC4373539

[16] Brandt C, Nolte H, Henschke S, Engström Ruud L, Awazawa M, Morgan DA, et al. Food perception primes hepatic ER homeostasis via melanocortin-dependent control of mTOR activation. Cell. 2018;175:1321–e133520. 30445039 10.1016/j.cell.2018.10.015 PMC6541012

[17] Henschke S, Nolte H, Magoley J, Kleele T, Brandt C, Hausen AC, et al. Food perception promotes phosphorylation of MFFS131 and mitochondrial fragmentation in liver. Science. 2024;384:438–46. 38662831 10.1126/science.adk1005

[18] Koch M, Varela L, Kim JG, Kim JD, Hernandez F, Simonds SE, et al. Hypothalamic POMC neurons promote cannabinoid-induced feeding. Nature. 2015;519:45–50. 25707796 10.1038/nature14260 PMC4496586

[19] Minère M, Wilhelms H, Kuzmanovic B, Lundh S, Fusca D, Claßen A, et al. Thalamic opioids from POMC satiety neurons switch on sugar appetite. Science. 2025;387:750–8. 39946455 10.1126/science.adp1510

[20] Navarro S, Soletto L, Puchol S, Rotllant J, Soengas JL, Cerdá-Reverter JM. 60 YEARS OF POMC: POMC: an evolutionary perspective. J Mol Endocrinol. 2016;56:T113–8. 26671895 10.1530/JME-15-0288

[21] Piazza PV, Cota D, Marsicano G. The CB1 receptor as the cornerstone of exostasis. Neuron. 2017;93:1252–74. 28334603 10.1016/j.neuron.2017.02.002

[22] Campbell JN, Macosko EZ, Fenselau H, Pers TH, Lyubetskaya A, Tenen D, et al. A molecular census of arcuate hypothalamus and median eminence cell types. Nat Neurosci. 2017;20:484–96. 28166221 10.1038/nn.4495 PMC5323293

[23] Henry FE, Sugino K, Tozer A, Branco T, Sternson SM. Cell type-specific transcriptomics of hypothalamic energy-sensing neuron responses to weight-loss. eLife. 2015;4:e09800. 10.7554/eLife.09800 PMC4595745 26329458

[24] Lam BYH, Cimino I, Polex-Wolf J, Nicole Kohnke S, Rimmington D, Iyemere V, et al. Heterogeneity of hypothalamic pro-opiomelanocortin-expressing neurons revealed by single-cell RNA sequencing. Mol Metab. 2017;6:383–92. 28462073 10.1016/j.molmet.2017.02.007 PMC5404100

[25] Chrétien M, Benjannet S, Gossard F, Gianoulakis C, Crine P, Lis M, et al. From beta-lipotropin to beta-endorphin and pro-opio-melanocortin. Can J Biochem. 1979;57:1111–21. 228824 10.1139/o79-143

[26] Nakanishi S, Inoue A, Kita T, Nakamura M, Chang AC, Cohen SN, et al. Nucleotide sequence of cloned cDNA for bovine corticotropin-beta-lipotropin precursor. Nature. 1979;278:423–7. 221818 10.1038/278423a0

[27] Panksepp J, Reilly P, Bishop P, Meeker RB, Vilberg TR, Kastin AJ. Effects of α-MSH on motivation, vigilance and brain respiration. Pharmacol Biochem Behav. 1976;5:59–64. 1013171 10.1016/0091-3057(76)90329-4

[28] Mountjoy KG, Robbins LS, Mortrud MT, Cone RD. The cloning of a family of genes that encode the melanocortin receptors. Science. 1992;257:1248–51. 1325670 10.1126/science.1325670

[29] Hruby VJ, Lu D, Sharma SD, Castrucci AL, Kesterson RA, al-Obeidi FA, et al. Cyclic lactam alpha-melanotropin analogues of Ac-Nle4-cyclo[Asp5, D-Phe7,Lys10] alpha-melanocyte-stimulating hormone-(4–10)-NH2 with bulky aromatic amino acids at position 7 show high antagonist potency and selectivity at specific melanocortin receptors. J Med Chem. 1995;38:3454–61. 7658432 10.1021/jm00018a005

[30] Kievit P, Halem H, Marks DL, Dong JZ, Glavas MM, Sinnayah P, et al. Chronic treatment with a melanocortin-4 receptor agonist causes weight loss, reduces insulin resistance, and improves cardiovascular function in diet-induced obese rhesus macaques. Diabetes. 2013;62:490–7. 23048186 10.2337/db12-0598 PMC3554387

[31] Fan W, Boston BA, Kesterson RA, Hruby VJ, Cone RD. Role of melanocortinergic neurons in feeding and the agouti obesity syndrome. Nature. 1997;385:165–8. 8990120 10.1038/385165a0

[32] Kim M-S, Rossi M, Abbott CR, AlAhmed SH, Smith DM, Bloom SR. Sustained orexigenic effect of agouti related protein may be not mediated by the melanocortin 4 receptor. Peptides. 2002;23:1069–76. 12126733 10.1016/s0196-9781(02)00039-6

[33] Li H, Xu Y, Jiang Y, Jiang Z, Otiz-Guzman J, Morrill JC, et al. The melanocortin action is biased toward protection from weight loss in mice. Nat Commun. 2023;14:2200. 37069175 10.1038/s41467-023-37912-z PMC10110624

[34] Greenman Y, Kuperman Y, Drori Y, Asa SL, Navon I, Forkosh O, et al. Postnatal ablation of POMC neurons induces an obese phenotype characterized by decreased food intake and enhanced anxiety-like behavior. Mol Endocrinol. 2013;27:1091–102. 23676213 10.1210/me.2012-1344 PMC5415244

[35] Rother E, Buch T, Brüning JC. Selective ablation of hypothalamic POMC-expressing neurons leads to hyperphagia and weight gain. Diabetol Stoffwechs. 2006;1:A62.

[36] Aponte Y, Atasoy D, Sternson SM. AGRP neurons are sufficient to orchestrate feeding behavior rapidly and without training. Nat Neurosci. 2011;14:351–5. 21209617 10.1038/nn.2739 PMC3049940

[37] Richardson J, Cruz MT, Majumdar U, Lewin A, Kingsbury KA, Dezfuli G, et al. Melanocortin signaling in the brainstem influences vagal outflow to the stomach. J Neurosci. 2013;33:13286–99. 23946387 10.1523/JNEUROSCI.0780-13.2013 PMC3742919

[38] Campos CA, Shiina H, Ritter RC. Central vagal afferent endings mediate reduction of food intake by Melanocortin-3/4 receptor agonist. J Neurosci. 2014;34:12636–45. 25232103 10.1523/JNEUROSCI.1121-14.2014 PMC4166153

[39] Fan W, Ellacott KLJ, Halatchev IG, Takahashi K, Yu P, Cone RD. Cholecystokinin-mediated suppression of feeding involves the brainstem melanocortin system. Nat Neurosci. 2004;7:335–6. 15034587 10.1038/nn1214

[40] Sohn J-W, Williams KW. Functional heterogeneity of arcuate nucleus pro-opiomelanocortin neurons: implications for diverging melanocortin pathways. Mol Neurobiol. 2012;45:225–33. 22328135 10.1007/s12035-012-8240-6 PMC3755591

[41] Yu H, Rubinstein M, Low MJ. Developmental single-cell transcriptomics of hypothalamic POMC neurons reveal the genetic trajectories of multiple neuropeptidergic phenotypes. eLife. 2022;11:e72883. 35044906 10.7554/eLife.72883 PMC8806186

[42] Fekete C, Zséli G, Singru PS, Kádár A, Wittmann G, Füzesi T, et al. Activation of anorexigenic pro-opiomelanocortin neurones during refeeding is independent of vagal and brainstem inputs. J Neuroendocrinol. 2012;24:1423–31. 22734660 10.1111/j.1365-2826.2012.02354.x

[43] Wu Q, Boyle MP, Palmiter RD. Loss of GABAergic signaling by AgRP neurons to the parabrachial nucleus leads to starvation. Cell. 2009;137:1225–34. 19563755 10.1016/j.cell.2009.04.022 PMC2729323

[44] Quarta C, Fisette A, Xu Y, Colldén G, Legutko B, Tseng Y-T, et al. Functional identity of hypothalamic melanocortin neurons depends on Tbx3. Nat Metab. 2019;1:222–35. 32694784 10.1038/s42255-018-0028-1 PMC8291379

[45] Knight ZA, Tan K, Birsoy K, Schmidt S, Garrison JL, Wysocki RW, et al. Molecular profiling of activated neurons by phosphorylated ribosome capture. Cell. 2012;151:1126–37. 23178128 10.1016/j.cell.2012.10.039 PMC3839252

[46] Leon S, Simon V, Lee TH, Steuernagel L, Clark S, Biglari N, et al. Single cell tracing of pomc neurons reveals recruitment of ‘ghost’ subtypes with atypical identity in a mouse model of obesity. Nat Commun. 2024;15:3443. 38658557 10.1038/s41467-024-47877-2 PMC11043070

[47] Hughes AC, Pittman BG, Xu B, Gammons JW, Webb CM, Nolen HG, et al. A single-vector intersectional AAV strategy for interrogating cellular diversity and brain function. Nat Neurosci. 2024;27:1400–10. 38802592 10.1038/s41593-024-01659-7 PMC12955835

[48] Biglari N, Gaziano I, Schumacher J, Radermacher J, Paeger L, Klemm P, et al. Functionally distinct POMC-expressing neuron subpopulations in hypothalamus revealed by intersectional targeting. Nat Neurosci. 2021;24:913–29. 34002087 10.1038/s41593-021-00854-0 PMC8249241

[49] Sabatini PV, Wang J, Rupp AC, Affinati AH, Flak JN, Li C, et al. tTARGIT AAVs mediate the sensitive and flexible manipulation of intersectional neuronal populations in mice. eLife. 2021;10:e66835. 33704065 10.7554/eLife.66835 PMC8026215

[50] Gaziano I, Corneliussen S, Biglari N, Neuhaus R, Shen L, Sotelo-Hitschfeld T, et al. Dopamine-inhibited POMCDrd2 + neurons in the ARC acutely regulate feeding and body temperature. JCI Insight. 2022;7:e162753. 36345942 10.1172/jci.insight.162753 PMC9675440

[51] Nicolas JC, Lee TH, Quarta C. Can brain neurons change identity? Lessons from obesity. Trends Endocrinol Metab. 2025;36:699-709. 10.1016/j.tem.2024.11.006 39643545

[52] Hentges ST, Otero-Corchon V, Pennock RL, King CM, Low MJ. Proopiomelanocortin expression in both GABA and glutamate neurons. J Neurosci. 2009;29:13684–90. 19864580 10.1523/JNEUROSCI.3770-09.2009 PMC2785088

[53] Saucisse N, Mazier W, Simon V, Binder E, Catania C, Bellocchio L, et al. Functional heterogeneity of POMC neurons relies on mTORC1 signaling. Cell Rep. 2021;37:109800. 34644574 10.1016/j.celrep.2021.109800

[54] Tadross JA, Steuernagel L, Dowsett GKC, Kentistou KA, Lundh S, Porniece M, et al. A comprehensive spatio-cellular map of the human hypothalamus. Nature. 2025;639:708–16. 39910307 10.1038/s41586-024-08504-8 PMC11922758

[55] Faouzi M, Leshan R, Björnholm M, Hennessey T, Jones J, Münzberg H. Differential accessibility of circulating leptin to individual hypothalamic sites. Endocrinology. 2007;148:5414–23. 17690165 10.1210/en.2007-0655

[56] Williams KW, Margatho LO, Lee CE, Choi M, Lee S, Scott MM, et al. Segregation of acute leptin and insulin effects in distinct populations of arcuate proopiomelanocortin neurons. J Neurosci. 2010;30:2472–9. 20164331 10.1523/JNEUROSCI.3118-09.2010 PMC2836776

[57] Mirzadeh Z, Alonge KM, Cabrales E, Herranz-Pérez V, Scarlett JM, Brown JM, et al. Perineuronal net formation during the critical period for neuronal maturation in the hypothalamic arcuate nucleus. Nat Metab. 2019;1:212–21. 31245789 10.1038/s42255-018-0029-0 PMC6594569

[58] Kohnke S, Buller S, Nuzzaci D, Ridley K, Lam B, Pivonkova H, et al. Nutritional regulation of oligodendrocyte differentiation regulates perineuronal net remodeling in the median eminence. Cell Rep. 2021;36:109362. 34260928 10.1016/j.celrep.2021.109362 PMC8293628

[59] Alonge KM, Mirzadeh Z, Scarlett JM, Logsdon AF, Brown JM, Cabrales E, et al. Hypothalamic perineuronal net assembly is required for sustained diabetes remission induced by fibroblast growth factor 1 in rats. Nat Metab. 2020;2:1025–33. 32895577 10.1038/s42255-020-00275-6 PMC7572652

[60] Beddows CA, Shi F, Horton AL, Dalal S, Zhang P, Ling C-C, et al. Pathogenic hypothalamic extracellular matrix promotes metabolic disease. Nature. 2024;633:914–22. 39294371 10.1038/s41586-024-07922-y PMC11424483

[61] Kuczynski-Noyau L, Karmann S, Alberton P, Martinez-Corral I, Nampoothiri S, Sauvé F, et al. A plastic Aggrecan barrier modulated by peripheral energy state gates metabolic signal access to arcuate neurons. Nat Commun. 2024;15:6701. 39112471 10.1038/s41467-024-50798-9 PMC11306556

[62] Sun J, Wang X, Sun R, Xiao X, Wang Y, Peng Y, et al. Microglia shape AgRP neuron postnatal development via regulating perineuronal net plasticity. Mol Psychiatry. 2024;29:306–16. 38001338 10.1038/s41380-023-02326-2

[63] Metz MJ, Daimon CM, King CM, Rau AR, Hentges ST. Individual arcuate nucleus Proopiomelanocortin neurons project to select target sites. Am J Physiol Regul Integr Comp Physiol. 2021;321:R982–9. 34755553 10.1152/ajpregu.00169.2021 PMC8714814

[64] Baker RA, Herkenham M. Arcuate nucleus neurons that project to the hypothalamic paraventricular nucleus: neuropeptidergic identity and consequences of adrenalectomy on mRNA levels in the rat. J Comp Neurol. 1995;358:518–30. 7593746 10.1002/cne.903580405

[65] Cota D, Proulx K, Smith KAB, Kozma SC, Thomas G, Woods SC, et al. Hypothalamic mTOR Signal Regulates Food Intake Sci. 2006;312:927–30. 10.1126/science.1124147 16690869

[66] Hu J, Jiang L, Low MJ, Rui L. Glucose rapidly induces different forms of excitatory synaptic plasticity in hypothalamic POMC neurons. PLoS ONE. 2014;9:e105080. 25127258 10.1371/journal.pone.0105080 PMC4134273

[67] Gao Y, Yao T, Deng Z, Sohn J-W, Sun J, Huang Y, et al. Trpc5 mediates acute leptin and serotonin effects via Pomc neurons. Cell Rep. 2017;18:583–92. 28099839 10.1016/j.celrep.2016.12.072 PMC5324780

[68] Sohn J-W, Xu Y, Jones JE, Wickman K, Williams KW, Elmquist JK. Serotonin 2 c receptor activates a distinct population of arcuate pro-opiomelanocortin neurons via TRPC channels. Neuron. 2011;71:488–97. 21835345 10.1016/j.neuron.2011.06.012 PMC3184528

[69] Berglund ED, Liu C, Sohn J-W, Liu T, Kim MH, Lee CE, et al. Serotonin 2 c receptors in pro-opiomelanocortin neurons regulate energy and glucose homeostasis. J Clin Invest. 2013;123:5061–70. 24177424 10.1172/JCI70338 PMC3859401

[70] Caron A, Dungan Lemko HM, Castorena CM, Fujikawa T, Lee S, Lord CC, et al. POMC neurons expressing leptin receptors coordinate metabolic responses to fasting via suppression of leptin levels. eLife. 2018;7:e33710. 29528284 10.7554/eLife.33710 PMC5866097

[71] Lemus MB, Bayliss JA, Lockie SH, Santos VV, Reichenbach A, Stark R, et al. A stereological analysis of NPY, POMC, orexin, GFAP astrocyte, and Iba1 microglia cell number and volume in diet-induced obese male mice. Endocrinology. 2015;156:1701–13. 25742051 10.1210/en.2014-1961

[72] Toda C, Santoro A, Kim JD, Diano S. POMC neurons: from birth to death. Annu Rev Physiol. 2017;79:209–36. 28192062 10.1146/annurev-physiol-022516-034110 PMC5669621

[73] Padilla SL, Carmody JS, Zeltser LM. Pomc-expressing progenitors give rise to antagonistic neuronal populations in hypothalamic feeding circuits. Nat Med. 2010;16:403–5. 20348924 10.1038/nm.2126 PMC2854504

[74] Sanz E, Quintana A, Deem JD, Steiner RA, Palmiter RD, McKnight GS. Fertility-regulating Kiss1 neurons arise from hypothalamic POMC-expressing progenitors. J Neurosci. 2015;35:5549–56. 25855171 10.1523/JNEUROSCI.3614-14.2015 PMC4388920

[75] Pelling M, Anthwal N, McNay D, Gradwohl G, Leiter AB, Guillemot F, et al. Differential requirements for neurogenin 3 in the development of POMC and NPY neurons in the hypothalamus. Dev Biol. 2011;349:406–16. 21074524 10.1016/j.ydbio.2010.11.007

[76] Lee B, Kim J, An T, Kim S, Patel EM, Raber J, et al. Dlx1/2 and Otp coordinate the production of hypothalamic GHRH- and AgRP-neurons. Nat Commun. 2018;9:2026. 29795232 10.1038/s41467-018-04377-4 PMC5966420

[77] Croizier S, Park S, Maillard J, Bouret SG. Central Dicer-miR-103/107 controls developmental switch of POMC progenitors into NPY neurons and impacts glucose homeostasis. eLife. 2018;7:e40429. 30311908 10.7554/eLife.40429 PMC6203430

[78] Messina A, Langlet F, Chachlaki K, Roa J, Rasika S, Jouy N, et al. A microrna switch regulates the rise in hypothalamic GnRH production before puberty. Nat Neurosci. 2016;19:835–44. 27135215 10.1038/nn.4298

[79] Turney MK, Nicholson WE, Kovacs WJ. Gene expression phenotyping of an ACTH-producing small cell lung cancer line. Mol Cell Endocrinol. 2004;219:105–13. 15149732 10.1016/j.mce.2004.01.005

[80] Surbhi, Wittmann G, Low MJ, Lechan RM. Adult-born Proopiomelanocortin neurons derived from Rax-expressing precursors mitigate the metabolic effects of congenital hypothalamic proopiomelanocortin deficiency. Mol Metab. 2021;53:101312. 34329773 10.1016/j.molmet.2021.101312 PMC8383116

[81] Croizier S, Bouret SG. Molecular control of the development of hypothalamic neurons involved in metabolic regulation. J Chem Neuroanat. 2022;123:102117. 35680104 10.1016/j.jchemneu.2022.102117

[82] Lechner L, Opitz R, Silver MJ, Krabusch PM, Prentice AM, Field MS, et al. Early-set POMC methylation variability is accompanied by increased risk for obesity and is addressable by MC4R agonist treatment. Sci Transl Med. 2023;15:eadg1659. 37467315 10.1126/scitranslmed.adg1659

[83] Yu H, Chiang A, Rubinstein M, Low MJ. The homeodomain transcription factor Six3 regulates hypothalamic Pomc expression and its absence from POMC neurons induces hyperphagia and mild obesity in male mice. Mol Metab. 2024;87:101993. 39025297 10.1016/j.molmet.2024.101993 PMC11327434

[84] Huang Y, Wang A, Zhou W, Li B, Zhang L, Rudolf AM, et al. Maternal dietary fat during lactation shapes single nucleus transcriptomic profile of postnatal offspring hypothalamus in a sexually dimorphic manner in mice. Nat Commun. 2024;15:2382. 38493217 10.1038/s41467-024-46589-x PMC10944494

[85] Xu Y, Yang D, Wang L, Król E, Mazidi M, Li L, et al. Maternal high fat diet in lactation impacts hypothalamic neurogenesis and neurotrophic development, leading to later life susceptibility to obesity in male but not female mice. Adv Sci. 2023;10:2305472. 10.1002/advs.202305472 PMC10724448 37867217

[86] Matuszewska J, Nowacka-Woszuk J, Radziejewska A, Grzęda E, Pruszyńska-Oszmałek E, Dylewski Ł, et al. Maternal cafeteria diet influences Kisspeptin (Kiss1), Kisspeptin receptor(Gpr54), and Sirtuin (Sirt1) genes, hormonal and metabolic profiles, and reproductive functions in rat offspring in a sex-specific manner†. Biol Reprod. 2023;109:654–68. 37665248 10.1093/biolre/ioad101 PMC10651067

[87] Zhai L, Zhao J, Zhu Y, Liu Q, Niu W, Liu C, et al. Downregulation of leptin receptor and kisspeptin/GPR54 in the murine hypothalamus contributes to male hypogonadism caused by high-fat diet-induced obesity. Endocrine. 2018;62:195–206. 29948931 10.1007/s12020-018-1646-9

[88] Dearden L, Buller S, Furigo I, Fernandez-Twinn DS, Ozanne S. Maternal obesity causes fetal hypothalamic insulin resistance and disrupts development of hypothalamic feeding pathways. Mol Metab. 2020;42:101079. 32919096 10.1016/j.molmet.2020.101079 PMC7549144

[89] Park S, Belfoul AM, Rastelli M, Jang A, Monnoye M, Bae H et al. Maternal low-calorie sweetener consumption rewires hypothalamic melanocortin circuits via a gut microbial co-metabolite pathway. JCI Insight. 2023;8:e156397. 10.1172/jci.insight.156397 PMC10322686 37014702

[90] Colldén G, Caron E, Bouret SG. Neonatal leptin antagonism improves metabolic programming of postnatally overnourished mice. Int J Obes 2005. 2022;46:1138–44. 10.1038/s41366-022-01093-4 35173277

[91] Haddad-Tóvolli R, Altirriba J, Obri A, Sánchez EE, Chivite I, Milà-Guasch M, et al. Pro-opiomelanocortin (POMC) neuron translatome signatures underlying obesogenic gestational malprogramming in mice. Mol Metab. 2020;36:100963. 32283518 10.1016/j.molmet.2020.02.006 PMC7152705

[92] Vogt MC, Paeger L, Hess S, Steculorum SM, Awazawa M, Hampel B, et al. Neonatal insulin action impairs hypothalamic neurocircuit formation in response to maternal high fat feeding. Cell. 2014;156:495–509. 24462248 10.1016/j.cell.2014.01.008 PMC4101521

[93] Quarta C, Fioramonti X, Cota D. POMC neurons dysfunction in diet-induced metabolic disease: hallmark or mechanism of disease?? Neuroscience. 2020;447:3–14. 31689486 10.1016/j.neuroscience.2019.09.031

[94] Zhan C, Zhou J, Feng Q, Zhang J, Lin S, Bao J, et al. Acute and long-term suppression of feeding behavior by POMC neurons in the brainstem and hypothalamus, respectively. J Neurosci. 2013;33:3624–32. 23426689 10.1523/JNEUROSCI.2742-12.2013 PMC6619547

[95] Trotta M, Bello EP, Alsina R, Tavella MB, Ferrán JL, Rubinstein M, et al. Hypothalamic Pomc expression restricted to GABAergic neurons suppresses Npy overexpression and restores food intake in obese mice. Mol Metab. 2020;37:100985. 32311511 10.1016/j.molmet.2020.100985 PMC7292867

[96] Zhu C, Jiang Z, Xu Y, Cai Z-L, Jiang Q, Xu Y, et al. Profound and redundant functions of arcuate neurons in obesity development. Nat Metab. 2020;2:763–74. 32719538 10.1038/s42255-020-0229-2 PMC7687864

[97] Raposinho PD, White RB, Aubert ML. The melanocortin agonist Melanotan-II reduces the orexigenic and adipogenic effects of neuropeptide Y (NPY) but does not affect the NPY-driven suppressive effects on the gonadotropic and somatotropic axes in the male rat. J Neuroendocrinol. 2003;15:173–81. 12535159 10.1046/j.1365-2826.2003.00962.x

[98] Nogueiras R, Wiedmer P, Perez-Tilve D, Veyrat-Durebex C, Keogh JM, Sutton GM, et al. The central melanocortin system directly controls peripheral lipid metabolism. J Clin Invest. 2007;117:3475–88. 17885689 10.1172/JCI31743 PMC1978426

[99] Li Y, Zhu S, Du D, Li Q, Xie K, Chen L, et al. TLR4 in POMC neurons regulates thermogenesis in a sex-dependent manner. J Lipid Res. 2023;64:100368. 37028769 10.1016/j.jlr.2023.100368 PMC10205441

[100] Tang Q, Liu Q, Yang X, Wu T, Huang C, Zhang J, et al. Sirtuin 6 supra-physiological overexpression in hypothalamic pro-opiomelanocortin neurons promotes obesity via the hypothalamus-adipose axis. FASEB J Off Publ Fed Am Soc Exp Biol. 2021;35:e21408. 10.1096/fj.202002607 33583107

[101] Tang M, Zhang Y, Zhang R, Zhang Y, Zheng J, Wang D, et al. GPSM1 in POMC neurons impairs brown adipose tissue thermogenesis and provokes diet-induced obesity. Mol Metab. 2023;79:101839. 37979657 10.1016/j.molmet.2023.101839 PMC10698273

[102] Tang Q, Liu Q, Li J, Yan J, Jing X, Zhang J, et al. Manf in POMC neurons promotes brown adipose tissue thermogenesis and protects against diet-induced obesity. Diabetes. 2022;71:2344–59. 35972224 10.2337/db21-1128 PMC9630086

[103] Voss-Andreae A, Murphy JG, Ellacott KLJ, Stuart RC, Nillni EA, Cone RD, et al. Role of the central melanocortin circuitry in adaptive thermogenesis of brown adipose tissue. Endocrinology. 2007;148:1550–60. 17194736 10.1210/en.2006-1389

[104] Singh U, Jiang J, Saito K, Toth BA, Dickey JE, Rodeghiero SR, et al. Neuroanatomical organization and functional roles of PVN MC4R pathways in physiological and behavioral regulations. Mol Metab. 2021;55:101401. 34823066 10.1016/j.molmet.2021.101401 PMC8689242

[105] Skibicka KP, Grill HJ. Hypothalamic and hindbrain melanocortin receptors contribute to the feeding, thermogenic, and cardiovascular action of melanocortins. Endocrinology. 2009;150:5351–61. 19854868 10.1210/en.2009-0804 PMC2795709

[106] Chitravanshi VC, Kawabe K, Sapru HN. Stimulation of the hypothalamic arcuate nucleus increases brown adipose tissue nerve activity via hypothalamic paraventricular and dorsomedial nuclei. Am J Physiol - Heart Circ Physiol. 2016;311:H433–44. 27402666 10.1152/ajpheart.00176.2016 PMC5008654

[107] Berglund ED, Liu T, Kong X, Sohn J-W, Vong L, Deng Z, et al. Melanocortin 4 receptors in autonomic neurons regulate thermogenesis and glycemia. Nat Neurosci. 2014;17:911–3. 24908101 10.1038/nn.3737 PMC4090093

[108] Jiang X, Liu K, Luo P, Li Z, Xiao F, Jiang H, et al. Hypothalamic SLC7A14 accounts for aging-reduced lipolysis in white adipose tissue of male mice. Nat Commun. 2024;15:7948. 39261456 10.1038/s41467-024-52059-1 PMC11391058

[109] De Jonghe BC, Hayes MR, Banno R, Skibicka KP, Zimmer DJ, Bowen KA, et al. Deficiency of PTP1B in POMC neurons leads to alterations in energy balance and homeostatic response to cold exposure. Am J Physiol - Endocrinol Metab. 2011;300:E1002–11. 21406615 10.1152/ajpendo.00639.2010 PMC3118594

[110] Ste. Marie L, Miura GI, Marsh DJ, Yagaloff K, Palmiter RD. A metabolic defect promotes obesity in mice lacking melanocortin-4 receptors. Proc Natl Acad Sci U S A. 2000;97:12339–44. 11027312 10.1073/pnas.220409497 PMC17343

[111] Farooqi IS, Keogh JM, Yeo GSH, Lank EJ, Cheetham T, O’Rahilly S. Clinical spectrum of obesity and mutations in the melanocortin 4 receptor gene. N Engl J Med. 2003;348:1085–95. 12646665 10.1056/NEJMoa022050

[112] Wallis NJ, McClellan A, Mörseburg A, Kentistou KA, Jamaluddin A, Dowsett GKC, et al. Canine genome-wide association study identifies DENND1B as an obesity gene in dogs and humans. Science. 2025;387:eads2145. 40048553 10.1126/science.ads2145 PMC7618706

[113] Chen KY, Muniyappa R, Abel BS, Mullins KP, Staker P, Brychta RJ, et al. RM-493, a melanocortin-4 receptor (MC4R) agonist, increases resting energy expenditure in obese individuals. J Clin Endocrinol Metab. 2015;100:1639–45. 25675384 10.1210/jc.2014-4024 PMC4399297

[114] Zheng Y, Rajcsanyi LS, Peters T, Dempfle A, Wudy SA, Hebebrand J, et al. Evaluation of the MC3R gene pertaining to body weight and height regulation and puberty development. Sci Rep. 2023;13:10419. 37369769 10.1038/s41598-023-37344-1 PMC10300021

[115] Lembertas AV, Pérusse L, Chagnon YC, Fisler JS, Warden CH, Purcell-Huynh DA, et al. Identification of an obesity quantitative trait locus on mouse chromosome 2 and evidence of linkage to body fat and insulin on the human homologous region 20q. J Clin Invest. 1997;100:1240–7. 9276742 10.1172/JCI119637 PMC508301

[116] Lee JH, Reed DR, Li WD, Xu W, Joo EJ, Kilker RL, et al. Genome scan for human obesity and linkage to markers in 20q13. Am J Hum Genet. 1999;64:196–209. 9915959 10.1086/302195 PMC1377718

[117] Chen AS, Marsh DJ, Trumbauer ME, Frazier EG, Guan X-M, Yu H, et al. Inactivation of the mouse melanocortin-3 receptor results in increased fat mass and reduced lean body mass. Nat Genet. 2000;26:97–102. 10973258 10.1038/79254

[118] Butler AA, Kesterson RA, Khong K, Cullen MJ, Pelleymounter MA, Dekoning J, et al. A unique metabolic syndrome causes obesity in the melanocortin-3 receptor-deficient mouse. Endocrinology. 2000;141:3518–21. 10965927 10.1210/endo.141.9.7791

[119] Possa-Paranhos IC, Butts J, Pyszka E, Nelson C, Congdon S, Cho D, et al. Medial hypothalamic MC3R signalling regulates energy rheostasis in adult mice. J Physiol. 2025;603:379–410. 39718394 10.1113/JP286699 PMC11737543

[120] Ghamari-Langroudi M, Cakir I, Lippert RN, Sweeney P, Litt MJ, Ellacott KLJ, et al. Regulation of energy rheostasis by the melanocortin-3 receptor. Sci Adv. 2018;4:eaat0866. 30140740 10.1126/sciadv.aat0866 PMC6105298

[121] Sutton AK, Goforth PB, Gonzalez IE, Dell’Orco J, Pei H, Myers MG, et al. Melanocortin 3 receptor-expressing neurons in the ventromedial hypothalamus promote glucose disposal. Proc Natl Acad Sci U S A. 2021;118:e2103090118. 33827930 10.1073/pnas.2103090118 PMC8053962

[122] Xu Y, Berglund ED, Sohn J-W, Holland WL, Chuang J-C, Fukuda M, et al. 5-HT2CRs expressed by pro-opiomelanocortin neurons regulate insulin sensitivity in liver. Nat Neurosci. 2010;13:1457–9. 21037584 10.1038/nn.2664 PMC3059249

[123] Berglund ED, Vianna CR, Donato J, Kim MH, Chuang J-C, Lee CE, et al. Direct leptin action on POMC neurons regulates glucose homeostasis and hepatic insulin sensitivity in mice. J Clin Invest. 2012;122:1000–9. 22326958 10.1172/JCI59816 PMC3287225

[124] Perez-Tilve D, Hofmann SM, Basford J, Nogueiras R, Pfluger PT, Patterson JT, et al. Melanocortin signaling in the CNS directly regulates circulating cholesterol. Nat Neurosci. 2010;13:877–82. 20526334 10.1038/nn.2569 PMC3100172

[125] Jo Y-H, Su Y, Gutierrez-Juarez R, Chua S. Oleic acid directly regulates POMC neuron excitability in the hypothalamus. J Neurophysiol. 2009;101:2305–16. 19261705 10.1152/jn.91294.2008 PMC2681442

[126] Michael NJ, Watt MJ. Long chain fatty acids differentially regulate sub-populations of arcuate POMC and NPY neurons. Neuroscience. 2020;451:164–73. 33002557 10.1016/j.neuroscience.2020.09.045

[127] Huo L, Gamber K, Greeley S, Silva J, Huntoon N, Leng X-H, et al. Leptin-dependent control of glucose balance and locomotor activity by POMC neurons. Cell Metab. 2009;9:537–47. 19490908 10.1016/j.cmet.2009.05.003 PMC2730605

[128] Shin AC, Filatova N, Lindtner C, Chi T, Degann S, Oberlin D, et al. Insulin receptor signaling in POMC, but not agrp, neurons controls adipose tissue insulin action. Diabetes. 2017;66:1560–71. 28385803 10.2337/db16-1238 PMC5440019

[129] Hill JW, Elias CF, Fukuda M, Williams KW, Berglund ED, Holland WL, et al. Direct insulin and leptin action in pro-opiomelanocortin neurons is required for normal glucose homeostasis and fertility. Cell Metab. 2010;11:286–97. 20374961 10.1016/j.cmet.2010.03.002 PMC2854520

[130] Dodd GT, Decherf S, Loh K, Simonds SE, Wiede F, Balland E, et al. Leptin and insulin act on POMC neurons to promote the browning of white fat. Cell. 2015;160:88–104. 25594176 10.1016/j.cell.2014.12.022 PMC4453004

[131] Burmeister MA, Ayala JE, Smouse H, Landivar-Rocha A, Brown JD, Drucker DJ, et al. The hypothalamic glucagon-like peptide 1 receptor is sufficient but not necessary for the regulation of energy balance and glucose homeostasis in mice. Diabetes. 2017;66:372–84. 27908915 10.2337/db16-1102 PMC5248999

[132] López-Otín C, Blasco MA, Partridge L, Serrano M, Kroemer G. Hallmarks of aging: an expanding universe. Cell. 2023;186:243–78. 36599349 10.1016/j.cell.2022.11.001

[133] Palmer AK, Jensen MD. Metabolic changes in aging humans: current evidence and therapeutic strategies. J Clin Invest. 2022;132:e158451. 10.1172/JCI158451 PMC9374375 35968789

[134] Jin K, Yao Z, van Velthoven CTJ, Kaplan ES, Glattfelder K, Barlow ST, et al. Brain-wide cell-type-specific transcriptomic signatures of healthy ageing in mice. Nature. 2025;638:182–96. 39743592 10.1038/s41586-024-08350-8 PMC11798837

[135] Yang S-B, Tien A-C, Boddupalli G, Xu AW, Jan YN, Jan LY. Rapamycin ameliorates age-dependent obesity associated with increased mTOR signaling in hypothalamic POMC neurons. Neuron. 2012;75:425–36. 22884327 10.1016/j.neuron.2012.03.043 PMC3467009

[136] Li G, Zhang Y, Wilsey JT, Scarpace PJ. Hypothalamic pro-opiomelanocortin gene delivery ameliorates obesity and glucose intolerance in aged rats. Diabetologia. 2005;48:2376–85. 16205885 10.1007/s00125-005-1943-8

[137] Tan B, Hedbacker K, Kelly L, Zhang Z, Moura-Assis A, Luo J-D, et al. A cellular and molecular basis of leptin resistance. Cell Metab. 2025;37:723–e7416. 40043692 10.1016/j.cmet.2025.01.001

[138] Kaushik S, Arias E, Kwon H, Lopez NM, Athonvarangkul D, Sahu S, et al. Loss of autophagy in hypothalamic POMC neurons impairs lipolysis. EMBO Rep. 2012;13:258–65. 22249165 10.1038/embor.2011.260 PMC3323137

[139] Newton AJ, Hess S, Paeger L, Vogt MC, Fleming Lascano J, Nillni EA, et al. AgRP innervation onto POMC neurons increases with age and is accelerated with chronic high-fat feeding in male mice. Endocrinology. 2013;154:172–83. 23161869 10.1210/en.2012-1643 PMC3529372

[140] Hickey AKS, Krashes MJ. Integrating hunger with rival motivations. Trends Endocrinol Metab. 2020;31:495–507. 32387196 10.1016/j.tem.2020.04.006

[141] Barbano MF, Zhang S, Chen E, Espinoza O, Mohammad U, Alvarez-Bagnarol Y, et al. Lateral hypothalamic glutamatergic inputs to VTA glutamatergic neurons mediate prioritization of innate defensive behavior over feeding. Nat Commun. 2024;15:403. 38195566 10.1038/s41467-023-44633-w PMC10776608

[142] Salgado I, de Li A, Burnett C, Gonzalez CJ, Becker SR, Horvath JJ. Toggling between food-seeking and self-preservation behaviors via hypothalamic response networks. Neuron. 2023;111:2899–e29176. 37442130 10.1016/j.neuron.2023.06.006 PMC10528369

[143] Reinoß P, Ciglieri E, Minére M, Bremser S, Klein A, Löhr H, et al. Hypothalamic Pomc neurons innervate the spinal cord and modulate the excitability of premotor circuits. Curr Biol. 2020;30:4579–e45937. 32976803 10.1016/j.cub.2020.08.103 PMC8847999

[144] He Z, Gao Y, Alhadeff AL, Castorena CM, Huang Y, Lieu L, et al. Cellular and synaptic reorganization of arcuate npy/agrp and POMC neurons after exercise. Mol Metab. 2018;18:107–19. 30292523 10.1016/j.molmet.2018.08.011 PMC6308029

[145] Portillo B, Hwang E, Ajwani J, Grose K, Lieu L, Wallace B, et al. NMDA receptors in POMC neurons connect exercise with insulin sensitivity. Diabetes. 2024;73:1942–51. 39401391 10.2337/dbi24-0002 PMC11579548

[146] Kang GM, Min SH, Lee CH, Kim JY, Lim HS, Choi MJ, et al. Mitohormesis in hypothalamic POMC neurons mediates regular exercise-induced high-turnover metabolism. Cell Metab. 2021;33:334–e3496. 33535098 10.1016/j.cmet.2021.01.003 PMC7959183

[147] Tang H-D, Dong W-Y, Hu R, Huang J-Y, Huang Z-H, Xiong W, et al. A neural circuit for the suppression of feeding under persistent pain. Nat Metab. 2022;4:1746–55. 36443522 10.1038/s42255-022-00688-5

[148] Yau YHC, Potenza MN. Stress and eating behaviors. Minerva Endocrinol. 2013;38:255–67. 24126546 PMC4214609

[149] Lee EJ, Hanchate NK, Kondoh K, Tong APS, Kuang D, Spray A, et al. A psychological stressor conveyed by appetite-linked neurons. Sci Adv. 2020;6:eaay5366. 32206712 10.1126/sciadv.aay5366 PMC7080447

[150] Ha GE, Cheong E. Chronic restraint stress decreases the excitability of hypothalamic POMC neuron and increases food intake. Exp Neurobiol. 2021;30:375–86. 34983879 10.5607/en21037 PMC8752322

[151] Fang X, Chen Y, Wang J, Zhang Z, Bai Y, Denney K, et al. Increased intrinsic and synaptic excitability of hypothalamic POMC neurons underlies chronic stress-induced behavioral deficits. Mol Psychiatry. 2023;28:1365–82. 36473997 10.1038/s41380-022-01872-5 PMC10005948

[152] Liu J, Garza JC, Truong HV, Henschel J, Zhang W, Lu X-Y. The melanocortinergic pathway is rapidly recruited by emotional stress and contributes to stress-induced anorexia and anxiety-like behavior. Endocrinology. 2007;148:5531–40. 17673512 10.1210/en.2007-0745 PMC3708592

[153] Qu N, He Y, Wang C, Xu P, Yang Y, Cai X, et al. A POMC-originated circuit regulates stress-induced hypophagia, depression and anhedonia. Mol Psychiatry. 2020;25:1006–21. 31485012 10.1038/s41380-019-0506-1 PMC7056580

[154] Leyrer-Jackson JM, Hood LE, Olive MF. Alcohol consumption preferentially activates a subset of pro-opiomelanocortin (POMC) producing neurons targeting the amygdala. Neuropharmacology. 2021;195:108674. 34153315 10.1016/j.neuropharm.2021.108674 PMC8475285

[155] Gangisetty O, Chaudhary S, Palagani A, Sarkar DK. Transgenerational inheritance of fetal alcohol effects on Proopiomelanocortin gene expression and methylation, cortisol response to stress, and anxiety-like behaviors in offspring for three generations in rats: evidence for male germline transmission. PLoS ONE. 2022;17:e0263340. 35143549 10.1371/journal.pone.0263340 PMC8830645

[156] Zheng D, Bi X, Zhang T, Han C, Ma T, Wang L, et al. Epigenetic alterations of the promoter region of the POMC gene in adolescent depressive disorder patients with nonsuicidal Self-injury behaviors. Psychol Res Behav Manag. 2020;13:997–1008. 33235529 10.2147/PRBM.S272445 PMC7678717

[157] Chang HS, Won ES, Lee H-Y, Ham B-J, Kim Y-G, Lee M-S. The association of proopiomelanocortin polymorphisms with the risk of major depressive disorder and the response to antidepressants via interactions with stressful life events. J Neural Transm. 2015;122:59–68. 25448875 10.1007/s00702-014-1333-9

[158] Zhang L, Verwer RWH, van Heerikhuize J, Lucassen PJ, Nathanielsz PW, Hol EM, et al. Progesterone receptor distribution in the human hypothalamus and its association with suicide. Acta Neuropathol Commun. 2024;12:16. 38263257 10.1186/s40478-024-01733-y PMC10807127

[159] Mousavi SG, Bateni S, Maracy MR, Mardanian F, Mousavi SH. Recurrent suicide attempt and female hormones. Adv Biomed Res. 2014;3:201. 25337531 10.4103/2277-9175.142046 PMC4202501

[160] Fu X-L, Li X, Ji J-M, Wu H, Chen H-L. Blood hormones and suicidal behaviour: a systematic review and meta-analysis. Neurosci Biobehav Rev. 2022;139:104725. 35690122 10.1016/j.neubiorev.2022.104725

[161] Ramírez S, Haddad-Tóvolli R, Radosevic M, Toledo M, Pané A, Alcolea D, et al. Hypothalamic pregnenolone mediates recognition memory in the context of metabolic disorders. Cell Metab. 2022;34:269-284.e9 35108514 10.1016/j.cmet.2021.12.023 PMC8815774

[162] Vercruysse P, Vieau D, Blum D, Petersén Å, Dupuis L. Hypothalamic Alterations in Neurodegenerative Diseases and Their Relation to Abnormal Energy Metabolism. Front Mol Neurosci. 2018;11:2 29403354 10.3389/fnmol.2018.00002 PMC5780436

[163] Burnett CJ, Li C, Webber E, Tsaousidou E, Xue SY, Brüning JC, et al. Hunger-Driven Motivational State Competition. Neuron. 2016;92:187–201 27693254 10.1016/j.neuron.2016.08.032 PMC5082717

